# Pharmaceutical Activities of Theanine: A Phytochemical Nutrient

**DOI:** 10.1002/fsn3.70811

**Published:** 2025-08-13

**Authors:** Xuelan Chong, Jian Hou, Hua‐Feng He

**Affiliations:** ^1^ School of Pharmacy Jining Medical University Rizhao China; ^2^ Rizhao Agricultural Technology Service Center Rizhao China

**Keywords:** cognitive improvement, neuroprotection, stress management, theanine

## Abstract

Theanine, first isolated from tea ( *Camellia sinensis* (L.) O. Kuntze), has been recognized for its multiple pharmaceutical functions. Typically, theanine is known for its protective effect on neural disorders. In this review, the neuroprotective effects of theanine were summarized in aspects of stress management, cognitive improvement, and reversal of neural injury. Meanwhile, the pharmaceutical functions of theanine in areas such as anti‐inflammatory effects, metabolic intervention, antineoplastic effect, etc., were explored and elucidated. Subsequently, functional expansion, applications for special medical purposes, and clarification of the pharmacological and metabolic mechanism of theanine were proposed as prospects for future research. All in all, identifying more desirable applications and furthering the functional advantages of theanine were the purposes. A systematic understanding of theanine's effect on neural health, as well as the development of theanine‐related products for mental health, would be desired.

## Graphical abstract

The neuroprotective effects of theanine were summarized in aspect of stress management, cognitive improvement and reversal of neural injury. Pharmaceutical functions of theanine in areas such as anti‐inflammatory effects, metabolic intervention, antineoplastic effect, etc., were explored and elucidated. Functional expansion, applications for special medical purposes, and clarification for the pharmacological and metabolic mechanism of theanine were proposed as the prospect.

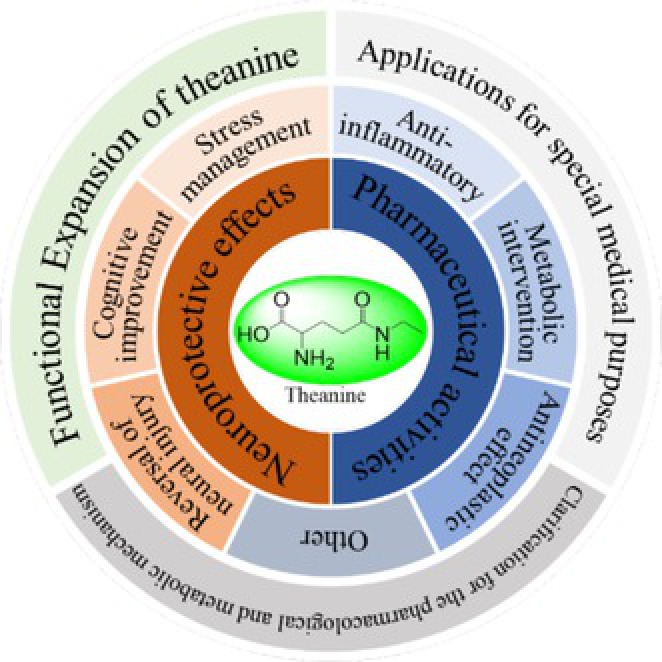

## 1 Introduction

Theanine, which is abundant in *Camellia sinensis* (L.) O. Kuntze specifically, has been found in several plants (Muszynska et al. 2020). Owing to its prominent contribution and substantial presence (accounting for 1.0% to 2.5% of the dry weight), _*L*_‐theanine stands as the primary factor responsible for the umami taste of tea (Zhang, Cao, Granato, et al. 2020; Rogers et al. 2007). Furthermore, it has become a consensus worldwide that drinking tea is beneficial for health (Muhammad et al. 2017). Apart from its spiritual and cultural connotations (Pan et al. 2022), the charm and prevalence of tea are attributed to its extensive health benefits, which are closely related to the antioxidant capacity of tea polyphenols (Bag et al. 2022; Jiang et al. 2022). In fact, there are amounts of physiologically active ingredients; theanine (Li, Liu, et al. 2022), caffeine, etc., make significant contributions (Rana et al. 2021; Payne et al. 2025).

**SCHEME 1:**
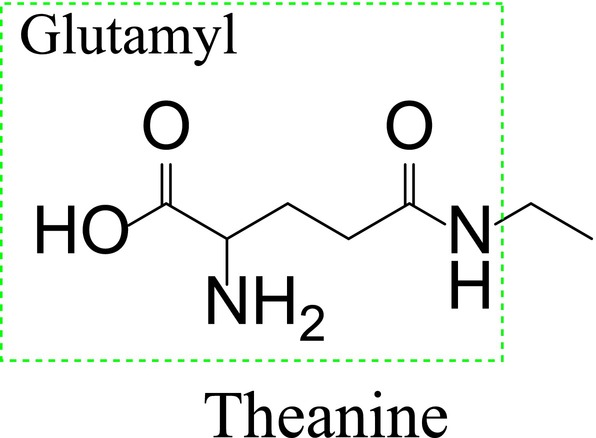
Chemical structure of theanine.

Being structurally homologous to glutamyl peptides (Scheme 1), it is facilitated for theanine to bind to receptor subtypes such as N‐methyl‐D‐aspartate (NMDA), kainic acid, and amino‐3‐hydroxy‐5‐methyl‐4‐isoxazole propionic acid (Wang, Brennan, et al. 2022). Although it does not participate in protein formation, theanine plays a collaborative role in the development of lateral roots (Chen, Lin, et al. 2023). To the delight of many, theanine is demonstrated to be biosynthesized in the root, but the leaf is the destination for accumulation, making it convenient for humans to consume theanine (Luo and He 2025).

Pioneering literatures had pronounced multiple health benefits of theanine. Herein, documents closely related to the pharmaceutical functions of theanine disclosed in recent years were reviewed and presented in aspect of neuroprotective effect and pharmaceutical activities. Thereby, functional expansion, application in special medical purposes, and clarification of the pharmacological mechanism were proposed as the prospect for research on theanine.

## 2 Neuroprotective Effects of Theanine

**SCHEME 2:**
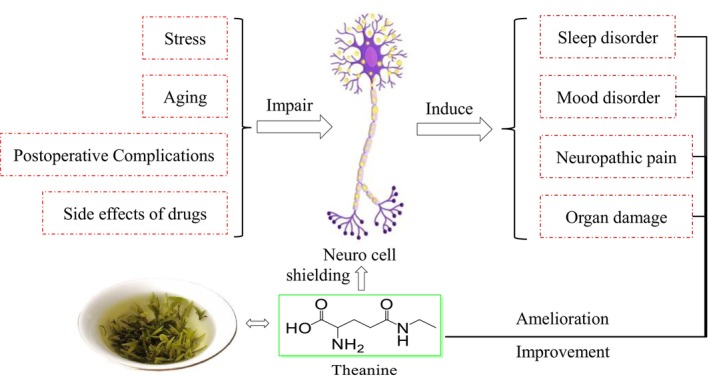
Participation of theanine in neuro‐related disorders.

With the accelerating pace of life, an increasing number of people are suffering from multifaceted stress. Neuropsychiatric disorders, e.g., sleep disorders, obsessive‐compulsive disorder (OCD), anxiety, depression, and psychotic symptoms, have emerged as significant threats. Coincidentally, aging as well as the chronic diseases it brings, e.g., cognitive disorders, are also a troublesome public health issue facing the world. Besides, complications after surgical operation as well as the side effects of drugs both could lead to pathological pain, even injury of organs. Pathologically, the abnormality of nerve cells is the root cause of these serious difficulties (Scheme 2). It has been demonstrated through amounts of documents that theanine could shield nerve cells from the pathological changes. Clinically, amelioration and improvement of theanine on sleep disorders, mood disorders, neuropathic pains, organ damage, and etc. were exhibited. The urgent need to develop lifestyle interventions for effectively managing mood disorders and ameliorating neurodegeneration has been highlighted as a critical issue. Compared to traditional drugs, nutrient combinations were generally considered healthier and more environmentally friendly in the treatment of mental disorders.

### 2.1 Stress Management

Traditionally, intake of tea would be beneficial for staying awake. However, a new insight that the balance between pathological sleep and energy levels under stress was proposed (Ouyang et al. 2022). It is indicated that tea consumption would be beneficial in regulating sleep quality, distinguishing from nervous excitement. Antagonistically, theanine could reduce the excitotoxicity of caffeine that affects sleep quality (Dasdelen et al. 2022; Baba et al. 2023). A positive effect on duration and habitual efficiency of sleep was observed with the administration of theanine by working adults (Thiagarajah et al. 2022). Changes in electrochemical activity in the brain, i.e., decreased electrocorticogram frequency, increased amplitude, and enhanced delta wave powers, were detected with the administration of theanine, as well as the Mg‐theanine complex (Dasdelen et al. 2022). Increasing of acetylcholine and γ‐aminobutyric acid (GABA), while decreasing of serotonin in the brain were found when administration of theanine synergistically with Neumentix, indicating the improvements in sleep disorders (Zhang, Jia, et al. 2021).

By increasing frontal region α power, a feature indicating relaxation in the brain, theanine exhibited a rapid and significant effect on individuals experiencing stress (Evans et al. 2021). As documented, theanine could cause metabolic changes in the hippocampus, which were believed to exhibit antidepressant effect (Unno et al. 2021). Similar to herbal medicine, theanine had been utilized as a dietary natural nutrient to manage mood disorders (Wu et al. 2022; Sarris et al. 2022; Borgonetti et al. 2020). Modulating the expression of Npas4 (Neuronal PAS Domain Protein 4), alleviation of the stress induced by group rearing was exhibited with the administration of theanine to mice (Unno et al. 2023). According to Yuan and co‐workers, the beneficial effects of theanine may be mediated by the enhancement of intracortical facilitation, involving NMDA receptor‐mediated processes, as well as by the attenuation of intracortical inhibition exerted by GABA receptor in the human primary motor cortex (Yuan et al. 2021). In clinical practice, added as a complementary therapy, theanine was proved to be both effective and tolerable (Shamabadi et al. 2023). A combination of cystine and theanine had been used in perioperative nutritional therapy to mitigate surgical stress (Tsuchiya and Kurihara 2022; Okamoto et al. 2022). The combination of theanine and walnut peptides exhibited universal activities, including antianxiety effects, neuroprotective properties, and memory enhancement (Zhao et al. 2023). In terms of attenuating nicotine‐withdrawal symptoms, which were similar to those of OCD, theanine exhibited positive potential effects (Alkhlaif et al. 2023). Furthermore, theanine played a crucial role in restoring the levels of potential biomarkers involved in various metabolic pathways to normal (Zhu et al. 2023).

### 2.2 Cognitive Improvement

A 1‐year observational study revealed that tea consumption was associated with a reduced risk of cognitive impairment among Chinese elderly individuals (Li, Yue, and Xiao 2022). In a survey of patients with mild cognitive impairments, tea consumption was shown to improve cognitive performance (Yin et al. 2023). The protective effect of theanine against isoflurane‐induced damage to neural stem cells indicated the potential to attenuate cognitive impairment (Chen, Lian, et al. 2020). Competitive inhibition of theanine on the cellular uptake of _*D*_‐serine was observed, which related to the hypofunction of receptors in the brain and a deficit in cognitive functions (Lakatos et al. 2020). As to chronic neurodegenerative diseases, e.g., Parkinson's disease (PD) (Raj et al. 2022), and Alzheimer's disease (AD) (Zeng et al. 2021), theanine presented noteworthy value (Luo et al. 2023). Rotenone‐induced neurotoxicity exhibited similar pathophysiological features to PD and had been employed as an animal model for decades. Treatment with theanine had shown evidence of ameliorating the behavioral and neurochemical dysfunction induced by rotenone (Chen, Wang, Soung, Chen, et al. 2022). Potentially suppressing the nuclear factor kappa‐light‐chain‐enhancer of activated B cells (NF‐κB) induced by lipopolysaccharide (LPS), theanine was found to reverse the motor deficits of PD. (Kumar et al. 2023) Theanine also up‐regulated the expression of silent information regulator 1, a new potential therapeutic target for AD (Zhang and Tang 2023). Collaborating with epigallocatechin gallate (EGCG), theanine could promote the repair and regeneration of nerve cells, which in turn could be applied to slow down the progression of AD (Xie et al. 2022). To treat and alleviate AD, chemicals originating from both sea and terrestrial sources were combined to provide therapy (Wang, Kong, et al. 2022). As representatives, theanine and nobiletin were selected to be combined with Antarctic krill oil, which exhibited a synergistic effect in ameliorating memory and cognition deficiencies.

Apart from no adverse effects being reported, the linear dose‐dependent improvement in attention warranted therapeutic applications of theanine (Dassanayake et al. 2022). Taking theanine as a base, a supplement was applied to children with Tourette syndrome and anxiety disorders, and a reduction in the severity of tic symptoms and anxiety symptoms was experienced (Rizzo et al. 2022). For attention deficit hyperactivity disorder, a potential therapeutic option involving theanine and caffeine was proposed (Kahathuduwa et al. 2020). Both originating from natural plants, memory enhancement was found in mice when treated with fructooligosaccharide and theanine, implying the potential to be developed as dietary supplements to promote human memory and cognition (Li, Jiang, et al. 2024).

### 2.3 Reversal of Neural Injury

The powerful neuroprotective effect of theanine endows it with a promising application in coping with the neurological damage caused by the adverse events (AEs) of drug administration. Generally, AEs in chemotherapy for cancer patients, including various nociceptive pains, would affect the therapeutic effect and acceptance. Amelioration of these neuropathies and neurotoxicities had been evidenced with the supplementation of theanine (Yang et al. 2023; Ye et al. 2023). By blocking the signaling pathways of glycogen synthase kinase 3 and Akt in the prefrontal cortex, pretreatment with theanine would assist in preventing the Δ‐9‐tetrahydrocannabinol induced chronic neuropathological adaptations (De Felice et al. 2021). Treatment with theanine ameliorated the neuropathic pain induced by chronic constriction injury of the sciatic nerve (Chen, Wang, Soung, Tseng, et al. 2022). Promotion of recovery of behavioral motor functions post‐spinal cord injury indicated potential use of theanine in motor dysfunction induced by neurogenic injury (Yang et al. 2020).

Functional injuries of organs could be caused by the chronic accumulation of stress. Ingestion of theanine could suppress the chronic stress induced brain atrophy (Unno et al. 2020). Daily consumption of theanine was beneficial in suppressing chronic sympathetic hyperactivity, which would be protective of bladder function (Matsuoka et al. 2021). By down‐regulating the heme oxygenase‐1 expression, theanine inhibited the neuron damage and apoptosis in the hippocampus to alleviate the neurological damage induced by ischemia/reperfusion injury in stroke (Zhao et al. 2020). Dose‐dependently, theanine inhibited liver and kidney injury induced by sepsis, a dysregulated host response to infection (Malkoc et al. 2020). Under a high protein diet, theanine regulated the content of neurotransmitters, exerting the potential to protect the liver and brain (Xu et al. 2023). Alleviation of acute alcoholic liver injury was proclaimed by theanine by down‐regulating the tumor necrosis factor‐α/NF‐κB signaling pathway (Sun et al. 2021). Treatment with theanine could shield the mice from reactive oxygen species induced renal fibrosis and inflammation (Xia et al. 2024). Combining transcriptomics and metabolomics, Fang and co‐workers evidenced the enhancement of serum biochemical indicators and the reduction of inflammatory cytokine levels in serum and cardiac tissues with the supplement of theanine, leading to the reversal of cardiac injury induced by obesity (Fang, Shen, Wang, et al. 2024). In the field of cell therapy, healing effects on injured liver tissue were exhibited by mesenchymal stem cells pre‐treated with theanine (Lai et al. 2022). Moreover, theanine exhibited a protective effect against acute radiation injury on the small intestine and bone marrow and improved the survival rate (Mstsuu‐Matsuyama et al. 2020; Matsuu‐Matsuyama et al. 2023). A neutralizing effect was shown by theanine in response to high dose EGCG‐induced acute liver injury (Peng, Dai, et al. 2021; Zhu et al. 2024).

## 3 Pharmaceutical Activities of Theanine

### 3.1 Anti‐Inflammatory

When it turns to inflammatory damage, theanine also exhibits protective effects. Accordingly, theanine could ameliorate dextran sulfate sodium‐induced pathological damage, block the expression of proinflammatory cytokines, and restore the balance of intestinal barrier disruption, making it an alternative for addressing colonic mucosa injury (Wang et al. 2020). By inhibiting the TLR4/p38 mitogen‐activated protein kinase (MAPK)/NF‐κB signaling pathway, theanine attenuated intestinal mucosa inflammation and improved the expression of tight junction protein (Chen, Chen, et al. 2023; Li, Huang, et al. 2024). Overall, amelioration of intestinal barrier functions was authorized to theanine (Wang et al. 2024). Similarly, theanine displayed protective effects in inflammatory bowel disease by inhibiting the progression of inflammation in intestinal tissues (Chen, Xiao, et al. 2020). In prevention of osteoarthritis, theanine decreased inflammatory cytokines and protected against extracellular matrix degradation (Bai et al. 1988). As well, theanine down‐regulated the production of interleukin‐23 and chemokines, which drive the pathogenesis of psoriasis (Xu et al. 2021). In alleviating 5‐fluorouracil‐induced intestinal mucositis and diarrhea, theanine was also effective (Yoneda et al. 2021).

### 3.2 Metabolic Intervention

Regulating signaling pathways, theanine could participate in the metabolism of glucose, lipid, and protein (Lin et al. 2020). Oral administration of theanine could promote fat browning via up‐regulating the expression of thermogenic genes (He et al. 2021). Consequently, an improvement in thermogenesis was evidenced in the case of diet‐induced obesity. Intraperitoneal treatment with theanine could not only enhance adaptive thermogenesis but also increase energy expenditure, inducing the browning of inguinal white adipose tissue (Peng, Xiao, et al. 2021). Theanine prevented colonic damage and obesity induced by a high‐fat diet by suppressing the phosphorylation of important proteins in the NF‐κB/MAPK pathways and regulating the metabolism of short‐chain fatty acids (Fang, Shen, Ma, et al. 2024; Xu et al. 2020). Meanwhile, dysbiosis of gut microbiota would be mitigated. Based on these results, theanine's effect on intestinal mucosal immunity was demonstrated. Enhancement of the stabilization of intestinal and the metabolism of glutamine would be obtained with the presence of theanine (Liu, Gong, et al. 2020). A nutraceutical supplement with theanine as the fundamental component was proven effective in modulating the fecal microbiome, thereby regulating anxiety and stress‐triggered functional gastrointestinal disorders (Cannas et al. 2021). By acting on amino acid transporters, theanine influenced the absorption of amino acids in the intestine (Liu et al. 2023). Additionally, supplementation of theanine was also useful in the improvement of lipid metabolism. Theanine had been documented to inhibit the formation of advanced glycation end products (AGEs), thereby maintaining homeostasis in the body and ameliorating liver aging (Saleh et al. 2019; Zeng et al. 2020). Proliferation of Sertoli cells and consumption of glucose could be prompted with exposure to a pharmacological dose of theanine (50 μM), indicating its potential to enhance male reproduction (Dias et al. 2019). Cooperating with EGCG, theanine promoted glycogen metabolism while suppressing the effect of EGCG on fatty acid and protein synthesis via adenosine 5′‐monophosphate (AMP)‐activated protein kinase signals (Lin et al. 2021).

### 3.3 Antineoplastic Effect

In vitro, ex vivo, and in vivo studies ascertained the participation of theanine in the treatment of various cancers (Shojaei‐Zarghani, Rafraf, and Yari‐Khoroushahi 2021). The intake of theanine would be associated with the amelioration of cancer cachexia, which was evidenced by the prevention of weight loss, internal fat loss, lower limb muscle loss, and an increase in serum interleukin‐6 levels (Kudamatsu et al. 2023). By inhibiting the extracellular signal‐regulated protein kinase/NF‐κB signaling pathway, theanine suppressed the transcription of matrix metalloproteinase 9 and snail. As a result, theanine may be considered a promising candidate for antimetastatic therapy of prostate cancer (Fan et al. 2021). By enhancing the expression of brain and muscle arnt‐like protein 1, a clock gene in melanoma cells, the viability of melanoma cells was reduced by theanine, along with the promoted apoptosis of melanoma cells and inhibition of migration (Zhang et al. 2022). In the case of dimethylhydrazine‐induced cancerous and precancerous lesions, the chemo‐preventive effect was displayed by theanine, which would be more pronounced when combined with theobromine (Shojaei‐Zarghani, Khosroushahi, and Rafraf 2021). Employing theanine as an adjuvant, liposomes were prepared with optimal sizes to sustain the release of doxorubicin and reduce adverse reactions in chemotherapy for malignant tumors (Zhao et al. 2019). A phase II trial indicated oral administration of cystine and theanine could reduce the incidence of AEs in chemotherapy, e.g., diarrhea, peripheral neuropathy (Hamaguchi et al. 2020; Kobayashi et al. 2020; Kawashiri et al. 2020).

### 3.4 Other Pharmaceutical Activities and Applications of Theanine

Additionally, as shown in Table 1, theanine has been found to exhibit various pharmaceutical activities beneficial to health (Maloh et al. 2022), including the prevention of cardiovascular diseases (CVDs) and immunomodulation. On the other hand, applications of theanine in the food industry and feed additives were documented. Even in fields such as contaminant degradation, bacterial infection control, nerve tissue engineering, and athletic performance enhancement, theanine plays a pivotal role.

**TABLE 1:** Other pharmaceutical activities and applications of theanine.

Function	Key finding	References
Prevention of CVDs	Theanine regulated scavenger receptor A at the translational level, inhibited the abnormal proliferation and migration of vascular smooth muscle cells	(Lei et al. 2021; Bi et al. 2020)
Immunomodulation	Theanine participated in adjusting immune function	(Chen, Kang, et al. 2023; Kikuchi et al. 2022; Ribeiro et al. 2020)
Feed additive	Dietary supplementation with theanine was helpful to improve meat quality and to neutralize oxidative stress, heat stress in poultry, cattle, Chinese mitten crabs ( *Eriocheir sinensis* )	(Dai et al. 2023; Muhammad et al. 2020; Alagawany et al. 2020; Zhang, Wang, Zhao, et al. 2020; Zhang, Wang, Chen, et al. 2020; Uyanga et al. 2022; Wang, Tang, et al. 2022; Wang et al. 2021; Yang et al. 2022, 2024; Muhammad, Xu, Zhang, et al. 2018; Muhammad, Xu, Faiz‐ul, et al. 2018)
Food industry	Addition of theanine was beneficial for storage of bacteria, development of resistant starch and ginkgo seed protein	(Kwon et al. 2025; Liu, Chen, et al. 2020; Hao et al. 2024; Zhang, Ge, et al. 2024)
Nerve tissue engineering	Theanine endowed graphene oxide the ability to withstand injury to the central nervous system	(Qi et al. 2020)
Bacterial infection control	A copper cluster equipped with theanine peptide exhibit outstanding antibacterial effects	(Zhang, Zhang, et al. 2021)
Degradation of contaminants	Theanine participated in the formation of iron‐based nanoparticles as sweeper to remove As(V). Reactive carbonyl species	(Wu et al. 2021; Zhong et al. 2023; Zhang, Yang, et al. 2024)
Athletic nutrition	Theanine enhanced shooting accuracy, cognitive function, and skeletal muscle fiber type transition in athletes	(Yilmaz et al. 2023; Chen, Zhang, Xue, Liang, et al. 2022; López‐Martínez et al. 2022)

## 4 Prospect for Future Research on Theanine

### 4.1 Functional Expansion of Theanine

Based on pioneer work, pharmaceutical functions of theanine had been demonstrated. However, as research advances and emerging health threats evolve, the multifaceted functions of theanine await systematic exploration. In addition to its intrinsic activities, the synergistic enhancement of theanine's efficacy when combined with other phytochemicals, particularly those coexisting in tea (Xia et al. 2022; Liu et al. 2022; Jia et al. 2022), warrants further investigation.

### 4.2 Applications of Theanine for Special Medical Purposes

Its potent neurorestorative properties endow theanine with boundless prospects for therapeutic development in neuroscience. Furthermore, no AEs and dosage limitations guarantee the dietary supplement of theanine. Listed among approved additives by the International Society of Sports Nutrition (Jagim et al. 2023), theanine demonstrates potential for development as a nutritional ergogenic aid for athletic performance enhancement.

### 4.3 Clarification for the Pharmacological and Metabolic Mechanism of Theanine

Although the neuropharmacological effects of theanine have been well‐characterized, particularly in neuroprotection and cognitive enhancement, its underlying molecular mechanisms and direct therapeutic targets require further investigation. Meanwhile, the in vivo metabolic dynamics of theanine, encompassing rapid intestinal absorption, extensive tissue distribution, enzymatic biotransformation pathways, and efficient renal excretion, remain to be elucidated.

Besides, the low content of metabolites, coupled with poor genetic stability and high costs, was all a disadvantage of endogenous theanine synthetase, hampering its use for industrial theanine production. Enzymatic recombination could be an alternative to address the low catalytic efficiency and specificity of natural enzymes.

## 5 Conclusion

Theanine has been shown to have various pharmaceutical functions, typically in neuroprotection including stress management, cognitive improvement, and reversal of nerval injury. Furthermore, pharmaceutical activities such as anti‐inflammatory, metabolic intervention, and antineoplastic effect, etc., were summarized. Moreover, future research on theanine is proposed to focus on functional expansion, applications for special medical purposes, and clarification for the pharmacological and metabolic mechanism of theanine. Additionally, microbial and enzyme engineering techniques would be proposed for large‐scale preparation of theanine. It is hoped that the advantages of theanine in the field of bioengineering and the pharmaceutical industry would continue to drive research efforts in this area. In the face of multiple threats to human health, theanine has the potential to contribute greener and more healthful solutions in the future.

## Author Contributions

**Xuelan Chong:** data curation (equal), investigation (supporting), writing – original draft (lead). **Jian Hou:** writing – review and editing (equal). **Hua‐Feng He:** conceptualization (lead), data curation (equal), supervision (lead), writing – review and editing (lead).

## Conflicts of Interest

The authors declare no conflicts of interest.

## Data Availability

The authors have nothing to report.
